# Cardiac troponin I levels in canine pyometra

**DOI:** 10.1186/1751-0147-49-6

**Published:** 2007-02-28

**Authors:** Ragnvi Hagman, Anne-Sofie Lagerstedt, Boel A Fransson, Annika Bergström, Jens Häggström

**Affiliations:** 1 Department of Small Animal Clinical Sciences, Faculty of Veterinary Medicine, Swedish University of Agricultural Sciences, Box 7037, SE-75007 Uppsala, Sweden; 2 Department of Veterinary Clinical Sciences, Washington State University, Pullman, WA, 99164-7060, USA

## Abstract

**Background:**

Myocardial injury may contribute to unexpected deaths due to pyometra. To detect myocardial damage, measurement of cardiac troponin I (cTnI) is currently the most sensitive and specific method. The aims of the present study were to evaluate presence of myocardial damage in canine pyometra by analysis of cTnI, to explore whether myocardial injury was associated with systemic inflammatory response syndrome (SIRS) and to evaluate whether other clinical or laboratory parameters were associated with cTnI increase.

**Methods:**

Preoperative plasma levels of cTnI were investigated in 58 female dogs with pyometra and 9 controls. The value of physical examination findings, haematological, serum biochemical and pro-inflammatory (CRP and TNF-α) parameters as possible predictors of increased cTnI levels was also evaluated.

**Results:**

Seven dogs with pyometra (12%) and one control dog (11%) had increased levels of cTnI. In the pyometra group, the levels ranged between 0.3–0.9 μg l^-1 ^and in the control dog the level was 0.3 μg l^-1^. The cTnI levels did not differ significantly between the two groups. No cardiac abnormalities were evident on preoperative physical examinations. Four of the pyometra patients died within two weeks of surgery, of which two were examined post mortem. In one of these cases (later diagnosed with myocarditis and disseminated bacterial infection) the cTnI levels increased from 0.9 μg l^-1 ^preoperatively to 180 μg l^-1 ^the following day when also heart arrhythmia was also detected. The other patient had cTnI levels of 0.7 μg l^-1 ^with no detectable heart pathology post mortem. CTnI increase was not associated with presence of SIRS. There was a trend for the association of cTnI increase with increased mortality. No preoperative physical examination findings and few but unspecific laboratory parameters were associated with increased cTnI levels.

**Conclusion:**

Increased cTnI levels were observed in 12% of the dogs with pyometra. The proportions of dogs with cTnI increase did not differ significantly in the pyometra group compared with the control group. CTnI increase was not associated with presence of SIRS. A trend for association of cTnI increase and mortality was observed. Preoperative physical examination findings and included laboratory parameters were poor predictors of increased cTnI levels.

## Background

Pyometra is a common reproductive disorder which affects nearly one fourth of all female dogs before they reach ten years of age [1]. The disease generates clinical signs associated with infection and inflammation in the uterus and may induce endotoxaemia and systemic inflammation with multiorgan effects [2,3]. Despite modern treatment routines the mortality rate due to pyometra is about 4% [1]. Myocardial injury secondary to endotoxaemia, inflammation, disseminated bacterial infection or infarcation, is suspected to be a contributing factor to unexpected deaths in female dogs with pyometra [4]. In dogs where electrocardiography, thoracic radiography, ultrasonography and laboratory parameters are normal, myocardial necrosis and ischemia may be difficult to detect clinically [5]. For definitive diagnosis heart muscle biopsy is often required, but it is not an acceptable procedure due to the risks and costs [5]. Measurement of cardiac troponin can be useful as a non-invasive method to determine suspected myocardial injury, for example in cases presented with cardiac arrhythmia, severe depression or multi organ dysfunctions.

Cardiac troponin I (cTnI) analysis is a highly sensitive and specific method for the detection of myocyte injury which has been used to diagnose myocardial damage in many mammalian species including humans, dogs and cats [6-8]. Troponins are intracellular contractile-regulating proteins in muscle cells and the isoenzyme cTnI is specific for heart muscle. Since even slightly increased levels of cTnI indicate myocyte injury, the analysis adds diagnostic and prognostic value to other clinical examinations in identifying patients at risk of cardiac events. The plasma levels of cTnI have been shown to be associated with the severity of myocardial injury and survival in dogs [9-11]. In humans, cTnI analysis is the standard biomarker for myocardial cell injury in heart disease [7]. In human patients cardiac troponin measurement has also been demonstrated to be able to predict mortality in septic, end-stage renal disease and acute stroke, and it is suggested to be a new mortality risk factor in intensive care units [7]. In addition, analysis of cTnI is valuable for monitoring treatment response since the concentrations directly correspond to the extent of injured myocard [12,13]. Measurement of cTnI may in the future be used for these diagnostic and prognostic purposes even in the canine species. In dogs, increased plasma levels of cTnI have been demonstrated in cases of toxaemia, babesiosis, myocarditis, and pericardial effusion and other heart diseases [10,11,14-16].

In canine pyometra, the role of inflammatory mediators in myocardial disease remains to be evaluated. Analysis of c-reactive protein (CRP) was reported to reliably predict myocardial damage, dysfunctions and outcome in human patients when combined with analysis of cTnI [7,12,17]. Troponin-positive human patients were demonstrated to have increased concentrations of tumour necrosis factor-α (TNF-α) compared with the troponin-negative ones [7,18]. The role of TNF-α in the pathogenesis of heart diseases is not completely understood [5,19].

It seems logical that dogs with pyometra and more severely affected general condition would also be at higher risk of developing myocardial injury. In a previous study conducted by our laboratory group the inflammatory mediator prostaglandin F_2α_, as measured in plasma by its main metabolite 15-keto-13,14-dihydro-PGF_2α _(PG-metabolite) was demonstrated to be increased in female dogs with pyometra and to be associated with outcome as measured by the length of hospitalisation [20]. In that study, which also presented the results of history questionnaires, clinical and laboratory findings, the PG-metabolite levels were significantly higher in pyometra cases positive for systemic inflammatory response syndrome (SIRS) compared with the SIRS-negative ones. Presence of SIRS has previously been linked with lower survival rates and longer hospitalisation [21]. Analysis of CRP has also shown promising predictive value for severity of pyometra [22]. The possible predictive value of cTnI analysis in dogs with pyometra remains to be determined.

The aim of the present study was to evaluate presence of myocardial injury by cTnI measurement in female dogs with pyometra. Furthermore we aimed to explore whether myocardial injury is more common in the patients with systemic inflammatory response syndrome (SIRS) and investigate the association of cTnI increase with other parameters (case history, mortality, hospitalisation length, physical examination findings, haematological and biochemical values, TNF-α, PG-metabolite and CRP concentrations). Detailed information on some of these variables in pyometra, although not all the same cases, is previously published in other studies [2,20,22].

## Methods

### Ethical approval

The study was approved by the Uppsala County Ethical Board, Tierp, Sweden, prior to onset of the clinical investigations.

### Animals

#### Pyometra group

In total 58 privately-owned bitches with the presumptive diagnosis of pyometra were included in the present study. The pyometra group consisted of 35 different breeds. All bitches were treated by ovariohysterectomy at the Department of Small Animal Clinical Sciences, Swedish University of Agricultural Studies, Uppsala, Sweden. The diagnosis was based on case history, physical examination and diagnostic imaging using ultrasonography and/or radiography to demonstrate an enlarged, fluid-filled uterus. The diagnosis was verified visually during the ovariohysterectomy and confirmed by histopathological examination at the Department of Pathology, National Veterinary Institute, Uppsala, Sweden. The admitting clinician filled out a form specifying rectal temperature, heart rate, respiratory rate, mucus membrane colour, capillary refill time, location for pain response at abdominal palpation, hydration status and general attitude at the time of admission. Postoperatively the heart rate, rectal temperature and respiratory rate were noted daily on a special form for as long as the patient was hospitalised. The heart was monitored by auscultation each day post surgery and if any signs of abnormalities were detected, additional electrocardiographic examinations and blood samplings for cTnI analysis were performed. Further information on treatment, complication to treatment and mortality were obtained from the medical records. None of the dogs had previously been medically treated for pyometra.

#### Control group

Healthy adult intact staff-owned female dogs (n = 9) were included in the control group which consisted of 6 different breeds. A history questionnaire was filled out, ensuring that the owner considered the bitch healthy for at least eight weeks prior to the examination. Complete physical examination, including heart and lung auscultation, was performed and documented by two of the authors who also filled out the form specifying rectal temperature, heart rate, respiratory rate, mucus membrane colour, capillary refill time, location for any pain response at abdominal palpation, hydration status and general attitude. None of the control bitches had previously been medically treated for pyometra, nor had suffered from any uterine disease at least one year after the study was finished.

### Blood collection and analyses

#### Pyometra group

Blood samples for biochemical, haematological and PG-metabolite analysis were obtained immediately before surgery in EDTA, sodium-heparinised and non-additive vacutainer tubes (Becton-Dickinson, Stockholm, Sweden). Biochemical and haematological analyses were performed using routine methods, at the Department of Biomedicine and Veterinary Public Health, Swedish University of Agricultural Sciences, Uppsala, Sweden. Analysed haematology parameters included packed cell volume (PCV), haemoglobin (Hb), white blood cell count (WBC), differential count of WBC including total count (BN) and percentage band neutrophils (PBN), nucleated erythrocytes, erythrocyte morphology, red blood cell mean corpuscular volume (MCV), red blood cell mean corpuscular haemoglobin concentration (MCHC) and platelet count (PC). Analysed serum biochemical parameters included alanine aminotransferase (ALAT), albumin, alkaline phosphatase (AP), blood urea nitrogen (BUN), cholesterol, glucose, total protein and the electrolytes sodium (Na), potassium (K), chloride (Cl) and calcium (Ca). For analysis of PG-metabolite, sodium heparinised plasma was assayed at the Department of Clinical Sciences, Swedish University of Agricultural Sciences, Uppsala, Sweden. A radioimmuno assay (RIA) was used to analyse 15-ketodihydro-PGF_2α _in duplicates [23]. The practical detection limit of the assay was 200 pmol l^-1^. Where necessary, dilution of the samples was performed to allow for accurate readings on the standard curve. Cardiac troponin I was analysed using a commercially available immunometrical method previously used in dogs for this purpose (Immulite, Troponin I, Diagnostic Products Corporation, Los Angeles, CA, USA) [13,14]. The detection limit for the analysis is 0.2 μg l^-1^, according to the manufacturer or even lower (0.1 μg l^-1^) according to a recent study [23]. Immediately after separation, the plasma from one of the EDTA tubes was stored in two aliquots at -70°C for TNF-α and CRP determinations. These plasma tubes were transported to Washington State University, Pullman, WA, USA, on dry ice and stored at -70°C until analysis. C-reactive protein was determined by a commercially available canine sandwich ELISA (Canine CRP ELISA kit, TriDelta Diagnostics Inc., Plains, NJ, USA). TNF-α concentrations were determined by a sandwich ELISA, kindly provided by Dr. WA Buurman (University of Maastricht, Maastricht, The Netherlands). This ELISA test utilising this antibody experimentally in dog plasma have shown a detection limit of 15 pg ml^-1^, which is 1/60 of the levels achieved after sub-lethal doses of endotoxin [26]. This ELISA test has shown a good correlation with the Walter and Eliza Hall Institute of Medical Research (WEHI) bioassay and is specific for biologically active TNF-α [26].

#### Control group

Blood samples from the control dogs were collected through the same procedure as for the diseased group after the physical examination.

### Histopathological examinations

The diagnosis of pyometra, according to the previously proposed definition was confirmed by gross and histopathological examination of hematoxylin-eosin stained sections of formaldehyde-fixated uteri and ovaries at the Department of Pathology, National Veterinary Institute (SVA), Uppsala, Sweden [27]. Samples for histopathological examinations were collected from several sites at both the uterine horns and body. The definition of pyometra was chronic purulent metritis, acute purulent metritis or purulent endometritis.

### Determination of a systemic inflammatory response

A patient was regarded as SIRS-positive if two or more of the following criteria were met: respiratory rate > 20 min^-1^; heart rate > 120 beats min^-1^; WBC < 6 or > 16 (× 10^9 ^l^-1^) or percentage band neutrophils (PBN)> 3%; temperature < 38.1 or > 39.2 [28]. The selected criteria have a high sensitivity (97%), but a low specificity (64%) in identifying SIRS [28]. The risk of misdiagnosing a true SIRS-positive dog (which could have serious consequences for that dog) is minor with the chosen criteria. Due to the low specificity, however, the SIRS-positive group will include 36% false-positive dogs.

### Statistical analyses

Statistical analyses were performed with the programme Statistica (Version 6.0, StatSoft Inc., Tulsa, USA). Spearman rank correlation coefficient (r_s_) was used to test for associations between interval-scale variables. Values below the detection limit, which in the data set occurred for cTnI, PG-metabolite, BN, segmented neutrophils, eosinophils, and basophils, were set to zero in the calculations. Mann-Whitney U-tests were used to test for differences in haematological and blood chemistry parameters between the control and pyometra group, and Fisher's exact test was used to test for association between detectable cTnI levels in dogs with SIRS or without SIRS, as tested in the pyometra group. Fisher's exact test was also used to test for association between detectable cTnI levels and survival as tested in the pyometra group. Significance was accepted at P < 0.05 for all statistical tests used in this study.

## Results

### Cardiac troponin I in bitches with pyometra

Detectable levels of cTnI were found in 7 (12%) of the 58 pyometra female dogs. The cTnI levels ranged between 0.3–0.9 μg l^-1 ^(Table 1). In one of the two female dogs (no.1) with the highest preoperative cTnI levels (0.9 μg l^-1^), another blood sample was obtained for cTnI analysis the following day, which demonstrated levels of 180 μg l^-1 ^(Table 1). That dog died within 24 hours after the last sample was obtained and autopsy was performed. The death was caused by disseminated bacterial infection with spread to various organs, including the heart where extensive myocarditis was demonstrated. The other pyometra case that was examined post mortem had preoperative cTnI values of 0.7 μg l^-1 ^and no pathological signs of myocardial disease. Two other female dogs died within 2 days and 2 weeks after surgery, respectively. The cTnI levels were not increased and because no post mortem examinations were performed in these cases, it is unclear whether these deaths were consequences of the pyometra.

**Table 1:** Data on selected parameters from the seven female dogs with pyometra (no. 1–7) with increased plasma levels of cardiac troponin I (cTnI).

Pyometra patient	cTnI (μg l^-1^)	CRP (mg l^-1^)	PGM (pmol l^-1^)	TNF-α (pg ml^-1^)	EPC (10^12 ^l^-1^)	PCV	Hb (g l^-1^)	WBC (10^9 ^l^-1^)	PBN (%)	SN (10^9 ^l^-1^)	Lymph (10^9 ^l^-1^)	Crea (μmol l^-1^)	ALAT (μkat l^-1^)	AP (μkat l^-1^)	BUN (mmol l^-1^)	Alb (g l^-1^)	Chol (mmol l^-1^)	Temp (°C)	HR (beats per minute)	Age (years)
1*	0.9	183	33500	0.00	154	0.4	152	4.5	20.0	3.1	0.1	257	5.6	6.4	18	18	5.4	40.4	120	13.6
2	0.9	285	3100	0.13	152	0.4	154	25.6	7.8	16.9	2.0	68	0.5	14.6	6.8	35	11.1	38.7	100	10.0
3*	0.7	-	432	-	165	0.5	165	16.6	7.2	13.3	1.0	75	0.9	4.1	7.6	35	11.9	39.1	100	11.8
4	0.6	300	7300	0.15	151	0.5	151	17.9	7.3	13.4	0.2	75	0.4	12.2	12	22	9.1	38.4	134	9.6
5	0.5	-	1244	-	-	-	-	-	-	-	-	256	0.6	9.2	23	37	14.1	39.9	164	7.1
6	0.3	369	2938	-	114	0.3	114	12.8	18.0	9.0	0.6	110	0.3	2.6	5.3	26	10.2	40.6	120	13.6
7	0.3	133	2532	0.15	119	0.3	119	16.4	18.3	11.6	1.1	50	1.1	11.6	2.9	28	6.0	39.0	160	9.5

The dogs were of the following different breeds: 1) German Shepherd, 2) Dachshund, 3) Leonberger, 4) Saluki, 5) Australian Kelpie, 6) Tibetian Spaniel, 7) Löwchen. Abbreviations used in the table: cTnI = cardiac troponin I; CRP = c-reactive protein; TNF-α = tumour necrosis factor α; PGM = prostaglandin F_2α _metabolite; EPC = red blood cell particle concentration; PCV = packed cell volume; Hb = haemoglobin; WBC = white blood cell count; PBN = percentage band neutrophils; SN = segmented neutrophils; Lymph = lymphocytes; Crea = creatinine; ALAT = alanine amino transferase; AP = alkaline phosphatase; BUN = blood urea nitrogen; Alb = albumin, Chol = cholesterol; Temp = body temperature.

* = died within two weeks of surgery.

### Cardiac troponin I in controls

The levels of cTnI were low or undetectable by means of the chosen method, in eight of the nine control female dogs. The remaining control dog (11%) had cTnI levels of 0.3 μg l^-1^.

### Cardiac troponin I and survival

There was a trend for increased mortality in the pyometra patients with detectable cTnI levels (Fisher's exact test, P = 0.067).

### Cardiac troponin I and SIRS

The cTnI concentrations did not differ significantly in the pyometra dogs with and without SIRS (Two-sample t-tests). Detectable cTnI levels were not associated with presence of SIRS (Fisher's exact test).

### Clinical findings, biochemistry and haematological parameters

The pyometra and control groups differed significantly (P < 0.05) regarding the PG-metabolite, TNF-α, and CRP levels, Hb, PCV, EPC, WBC, BN, PBN, neutrophils, lymphocytes, monocytes, PC, AP, albumin, cholesterol, potassium, body temperature, heart rate and age (Table 2).

**Table 2:** The table illustrates data on cardiac troponin I (cTnI) levels and selected parameters in the pyometra and control groups. P-values for the significance level, median and range values are shown (Mann-Whitney U-tests).

		Control		Pyometra	
	P-value (Mann-Whitney U-test)	Median (Range)	n	Median (Range)	n
cTnI^a ^(μg l^-1^)	0.949	0.00 (0.0–0.3)	9	0.00 (0.0–0.9)	58
TNF-α^b ^(pg ml^-1^)	0.060	0.005 (0.0–0.01)	2	0.14 (0.0–0.41)	33
CRP^c ^(mg l^-1^)	0.024	23.8 (19.6–28.0)	2	212.2 (26.4–369.0)	36
PGM^d ^(pmol l^-1^)	<0.001	0.0 (0–772)	9	3950 (0–33500)	58
lymphocytes (10^9 ^l^-1^)	<0.01	2.5 (0.1–5.0)	9	1.3 (0.0–3.9)	56
BUN^e ^(mmol l^-1^)	0.029	6.6 (3.5–16.3)	9	4.0 (1.6–23.1)	48
AP^f ^(μkat l^-1^)	<0.001	1.6 (1.0–3.2)	9	4.2 (0.4–34.0)	57
ALAT^g ^(μkat l^-1^)	0.077	0.60 (0.3–1.3)	9	0.30 (0.1–5.6)	57

^a^cTnI = cardiac troponin I; ^b^TNFα = tumour necrosis factor α; ^c^CRP = c-reactive protein; ^d^PGM = prostaglandin F_2α _metabolite; ^e^BUN = blood urea nitrogen; ^f^AP = alkaline phosphatase; ^g^ALAT = alanine amino transferase.

### Correlations between hospitalisation length, cardiac troponin I and other examined clinical and laboratory parameters

Hospitalisation length was not correlated with cTnI levels but with the following parameters: duration of illness before admission (r_s _= 0.295), body temperature (r_s _= 0.424), respiratory rate (r_s _= -0.315), age (r_s _= 0.302), TNF-α (r_s _= 0.345), PG (r_s _= 0.453), BN (r_s _= 0.413), PBN (r_s _= 0.397), segmented neutrophils (r_s _= 0.337), albumin (r_s _= -0.500), AP (r_s _= 0.451), cholesterol (r_s _= 0.353), monocytes (r_s _= 0.383), MCV (r_s _= -0.392), WBC (r_s _= 0.331), Hb (r_s _= -0.291), and PC (r_s _= -0.266).

### Correlations between cardiac troponin I and other examined clinical and laboratory parameters

Cardiac troponin I levels were significantly (P < 0.05) correlated with BUN (r_s _= 0.320), AP (r_s _= 0.282), ALAT (r_s _= 0.266) and lymphocytes (r_s _= -0.246).

## Discussion

In the present study, 7 of the 58 dogs with pyometra had increased plasma levels of cTnI. The levels ranged between 0.3–0.9 μg l^-1^, which indicates minor myocardial damage. Even slight elevations of cardiac troponin are specific for myocyte injury [5]. Unnoticed myocard injury, as measured by increased cTnI levels, is frequent in critically ill human patients and is associated with increased mortality rates [29]. The analysis thus adds diagnostic and prognostic value to other clinical examinations in identifying patients at risk for cardiac events [29]. In humans, events reported in the induction of myocard injury include reduction in oxygen supply in combination with increased myocard oxygen consumption or increasing wall stress, hypertension with left wall hypertrophy, tachycardia, infiltrative diseases, cardiac trauma, pulmonary embolism or myocardial toxins as a consequence of sepsis [29]. It is probable that the same types of events may induce myocard damage also in dogs. Reference values for plasma cTnI in healthy dogs has (in two different studies using the same method as in the present study [13,14]) previously been determined to be < 0.5 μg l^-1 ^and < 0.24 μg l^-1^. Four of the dogs with pyometra in the present study had cTnI levels over < 0.5 μg l^-1^, and all had cTnI levels above < 0.24 μg l^-1^.

Heart muscle injury occurs in female dogs with pyometra for several reasons. Bacteraemia, septicaemia and disseminated bacterial infection with possible subsequent myocarditis are not uncommon complications of the disease [30]. Pyometra is known to induce both endotoxaemia and SIRS, which may directly injure heart muscle cells [3,31,32]. Multiple organ dysfunctions and coagulopathies such as disseminated intravascular coagulation (DIC) are other severe complications that can induce myocard injury [21]. Further, the renal function is often negatively affected in dogs with pyometra which might lead to secondary uraemic myocardial damage [33-35]. In the present study 53% of the pyometra dogs were SIRS-positive, which is in the range of the proportion previously reported [2]. The hypothesis that more severely affected animals (SIRS-positive) would also be at higher risk of developing myocardial injury was rejected since cTnI increase was not associated with fulfilment of criteria for SIRS.

Cardiac troponin levels did not correlate with any of the inflammatory markers (PG-metabolite, CRP, TNF-α) examined in the present study. Measurement of these parameters was thus not useful to predict or identify dogs with increased cTnI levels despite previous reports of an association of TNF-α with cardiac troponin and the predictive value of CRP for cardiovascular events [7,36]. Both increased CRP and PG-metabolite levels have previously been associated with presence of SIRS in canine pyometra [20,22].

In the case where cardiac arrhythmia was discovered by auscultation on the first day post surgery, the cTnI levels were very high at that point in time (180 μg l^-1^), indicative of severe myocardial injury. That dog (German Shepherd, 13.6 years old) died later during the day and the increased level of cTnI was probably caused by the extensive myocarditis and disseminated bacterial infection demonstrated on subsequent post mortem gross and histopathological examination. The preoperative cTnI levels of 0.9 μg l^-1^indicate that additional myocardial injury developed after the first sample was obtained. No other concurrent cardiac disease was identified, which could otherwise account for the cTnI increase especially in elderly female dogs with pyometra. In cases with arrhythmia or thoracic radiograph abnormalities, it is valuable to rule in or out cardiac muscle injury in order to optimize the treatment. The cTnI analysis may also be functional in managing treatment response and healing since the plasma levels previously have been shown to be correlated with the severity of myocardial injury and survival [9-11]. Since the cTnI levels decrease to baseline within 5–10 days of the initial injury, it is important to obtain the blood samples as soon as myocardial injury is suspected [8].

Only one of the control dogs in the present study had detectable cTnI levels (0.3 μg l^-1^). In two previously published studies 51% and 19 % of the tested healthy control dogs had detectable levels of cTnI, as analysed by the same method. Since cTnI is intracellular it should generally not be present at all in the peripheral circulation unless some myocardial injury has occurred [5]. However, cTnI levels may increase minimally in healthy animals due to strenuous exercise or noncardiac disease in some cases [5]. That the control dog in the present study had cTnI level of 0.3 μg l^-1 ^might be due to minor damage of cardiac myocytes which did not affect the clinical appearance, haematological or biochemistry parameters. Since that dog was clinically healthy and not further examined, the exact cause for the cTnI increase remains unknown.

Measurement of cTnI levels in cases where an evaluation of the heart muscle status is indicated can be valuable for adjusting treatment and monitoring of the individual dog. This is relevant for example in pyometra dogs with concurrent heart disease or when myocard injury can be suspected. Myocyte damage, as measured by cTnI increase, was otherwise unpredictable by preoperative findings at physical examination and all the other laboratory parameters investigated in the present study. The cTnI concentrations correlated with BUN, AP, ALAT and negatively with lymphocytes. BUN, AP and ALAT generally reflect impaired kidney or liver function and hepatocyte damage and they are therefore poor predictors of myocyte injury.

However, troponin is cleared via the kidneys which explain why decreased kidney function may result in increased blood concentrations. Structural damage of proximal tubuli and some decrease in glomerular filtration rate have been demonstrated in dogs with pyometra [34,35]. Increased cTnI levels have also been demonstrated in azotaemic cats and dogs [37]. Still, cTnI analysis has been demonstrated to be useful in identifying myocardial injury in human renal failure patients without overt cardiac disease [38]. Concurrent structural cardiac disease could also induce increased cTnI values especially in elderly pyometra patients, but this was not suspected in any of the clinical cases due to the absence of heart murmurs. The cTnI levels were also not associated with increasing age. The clinical significance of minor cTnI increase still remains to be determined in dogs without clinical signs of cardiac dysfunction.

Potential life-threatening consequences of pyometra include endotoxaemia, bacteraemia, septicaemia, SIRS, DIC, multiple organ dysfunctions and disseminated bacterial infection of vital organs [2,21,29]. To evaluate the predictive value of cTnI for survival, an increased patient number is necessary since only two female dogs died of causes related to pyometra. In the second confirmed fatality, a dog with a thin-walled uterus which ruptured during surgery, the dog died within one hour after surgery was completed. This bitch had preoperative cTnI values of 0.7 μg l^-1 ^and no pathological signs of heart muscle disease on the autopsy (histopathological and gross examination). Of the remaining two fatalities one occurred two weeks after surgery, and the other one day after surgery, shortly after departure from the veterinary clinic. Whether these deaths were associated with complications of the pyometra or related to heart dysfunction is unclear since no autopsies were performed. In both these female dogs cTnI was not detected in the plasma preoperatively. The four pyometra patients that died ranged between 8.1–13.6 years of age. Two of the seven pyometra dogs with detectable cTnI levels died (28%) in comparison with two of the fifty-one pyometra dogs without cTnI increase (4%). A trend for the association of detectable cTnI levels with increased mortality was apparent when evaluated in the pyometra group (P = 0.067).

As for prognostic value, the cTnI levels were not correlated with the outcome as measured in this study by length of the hospitalisation. Pyometra cases overiohysterectomised at our clinic are generally dismissed 1–2 days after surgery. Additional complications lead to longer hospitalisation. Although a relatively crude measurement, length of hospitalisation has previously been used for this purpose in studies of human diseases [39] and in dogs [2,20].

## Conclusion

Minor myocardial injury was present in 12% of the female dogs with pyometra. The proportions of cases with increased cTnI levels did not differ significantly in the pyometra patient group when compared with healthy control dogs. Presence of SIRS was not associated with increased cTnI values. A trend for the association of detectable cTnI levels with increased mortality was apparent, as evaluated in the pyometra group. Findings at physical examination, haematological, biochemical and inflammatory parameters evaluated preoperatively in the present study, were all poor predictors of myocardial cell injury as determined by cTnI analysis.

## Competing interests

The author(s) declare that they have no competing interests.

## Authors' contributions

RH, ASL, BAF and AB were involved in the study design and collection of the samples. RH, AB and BAF were responsible for data acquisition. RH was responsible for data analysis and manuscript preparation. JH, ASL participated in the study design, and and revision of the manuscript. All authors read and approved the final manuscript.
